# Supplementary material for: Spatio-Temporal Variation of Gender-Specific Hypertension Risk: Evidence from China
Source: Int J Environ Res Public Health. 2019 Nov 17;16(22):4545. doi: 10.3390/ijerph16224545 (PMC6888411; doi:10.3390/ijerph16224545)
Supplement: Supplementary file 1 [file ijerph-16-04545-s001.pdf]

Table S1 OR of Age during 1991-2015

	year	OR(Mean)	OR(95% CI)
males	1991	1.001	(0.981,1.021)
	1993	1.011	(0.994,1.029)
	1997	**1.028**	(1.013,1.043)
	2000	**1.031**	(1.017,1.046)
	2004	**1.033**	(1.022,1.045)
	2006	**1.032**	(1.022,1.042)
	2009	**1.039**	(1.030,,1.048)
	2011	**1.033**	(1.025,,1.041)
	2015	**1.040**	(1.034,,1.047)
females	1991	1.010	(0.988,1.032)
	1993	1.017	(0.995,1.039)
	1997	**1.031**	(1.013,1.049)
	2000	**1.034**	(1.018,1.051)
	2004	**1.035**	(1.021,1.049)
	2006	**1.034**	(1.022,1.047)
	2009	**1.041**	(1.030,,1.053)
	2011	**1.036**	(1.025,,1.046)
	2015	**1.041**	(1.032,,1.050)

Table S2 OR of Overweight during 1991-2015

	year	OR(Mean)	OR(95% CI)
males	1991	**1.035**	(1.017,1.053)
	1993	**1.038**	(1.022,1.055)
	1997	**1.039**	(1.027,1.051)
	2000	**1.036**	(1.026,1.045)
	2004	**1.035**	(1.026,1.043)
	2006	**1.034**	(1.026,1.041)
	2009	**1.037**	(1.030,1.044)
	2011	**1.034**	(1.027,1.040)
	2015	**1.040**	(1.034,1.045)
females	1991	**1.018**	(1.002,1.038)
	1993	**1.021**	(1.006,1.040)
	1997	**1.028**	(1.017,1.043)
	2000	**1.028**	(1.018,1.041)
	2004	**1.029**	(1.019,1.041)
	2006	**1.029**	(1.020,1.041)
	2009	**1.035**	(1.026,1.046)
	2011	**1.031**	(1.024,1.041)
	2015	**1.036**	(1.029,1.045)

Table S3 OR of Alcohol Consumption during 1991-2015

	year	OR(Mean)	OR(95% CI)
males	1991	**0.978**	(0.970, 0.986)
	1993	**0.979**	(0.971, 0.987)
	1997	**0.985**	(0.976, 0.993)
	2000	**0.986**	(0.977, 0.994)
	2004	**0.989**	(0.981, 0.998)
	2006	**0.990**	(0.982, 0.999)
	2009	0.995	(0.987,1.003)
	2011	0.994	(0.986, 1.003)
	2015	1.003	(0.994, 1.012)
females	1991	**0.929**	(0.918, 0.941)
	1993	**0.936**	(0.924, 0.948)
	1997	**0.950**	(0.936, 0.964)
	2000	**0.962**	(0.949, 0.975)
	2004	**0.977**	(0.960, 0.995)
	2006	**0.983**	(0.967, 0.999)
	2009	**1.017**	(1.002,1.032)
	2011	1.010	(0.997, 1.023)
	2015	**1.065**	(1.042, 1.088)

Table S4 OR of Smoking during 1991-2015

	year	OR(Mean)	OR(95% CI)
males	1991	**1.014**	(1.005,1.024)
	1993	**1.017**	(1.007,1.027)
	1997	**1.024**	(1.013,1.034)
	2000	**1.025**	(1.015,1.036)
	2004	**1.026**	(1.016,1.036)
	2006	**1.027**	(1.017,1.037)
	2009	**1.031**	(1.022,1.041)
	2011	**1.031**	(1.021,1.041)
	2015	**1.044**	(1.033,1.055)
females	1991	**0.865**	(0.828, 0.902)
	1993	**0.886**	(0.849, 0.923)
	1997	**0.911**	(0.861, 0.962)
	2000	**0.938**	(0.883, 0.994)
	2004	0.985	(0.925, 1.047)
	2006	0.997	(0.928, 1.068)
	2009	**1.102**	(1.027,1.181)
	2011	**1.090**	(1.024, 1.157)
	2015	**1.444**	(1.273, 1.631)
